# Systemic Anticancer Treatment Near the End of Life: a Narrative Literature Review

**DOI:** 10.1007/s11864-023-01115-x

**Published:** 2023-07-27

**Authors:** Teresa Geyer, Nguyen-Son Le, Iris Groissenberger, Franziska Jutz, Lisa Tschurlovich, Gudrun Kreye

**Affiliations:** 1 Karl Landsteiner University of Health Sciences, Dr. Karl Dorrek-Straße 30, 3500 Krems an der Donau, Austria; 2 Division of Palliative Care, Department of Internal Medicine 2, Karl Landsteiner University of Health Sciences, University Hospital of Krems, Mitterweg 10, 3500 Krems an Der Donau, Austria

**Keywords:** Systemic anticancer treatment (SACT), End of life (EOL), Palliative care

## Abstract

Systemic anticancer therapy (SACT) includes different treatment modalities that can be effective in treating cancer. However, in the case of disease progression, cancers might become incurable and SACT might reach its limits. In the case of incurable cancers, SACT is often given in a palliative setting, with the goal of improving the patients’ quality of life (QOL) and their survival. In contrast, especially for patients who approach end of life (EOL), such treatments might do more harm than good. Patients receiving EOL anticancer treatments often experience belated palliative care referrals. The use of systemic chemotherapy in patients with advanced cancer and poor prognosis approaching the EOL has been associated with significant toxicity and worse QOL compared to best supportive care. Therefore, the American Society of Clinical Oncology (ASCO) has discouraged this practice, and it is considered a metric of low-value care by Choosing Wisely (Schnipper et al. in J Clin Oncol 4;30(14):1715-24). Recommendations of the European Society for Medical Oncology (ESMO) suggest that especially chemotherapy and immunotherapy should be avoided in the last few weeks of the patients’ lives. In this narrative review, we screened the current literature for the impact of SACT and factors predicting the use of SACT near the EOL with discussion on this topic.

## Introduction

Due to new developments regarding systemic anticancer treatment (SACT), patients with metastasized and inoperable or locally advanced and inoperable solid tumors may have a survival time of several years [1]. However, the course of the disease is hard to predict and can sometimes expand to several decades. The goal of antitumor therapy in advanced, metastatic cancer is usually not cure, but symptom control and prolongation of survival. A considerable percentage (20–50%) of patients diagnosed with advanced cancer undergo antitumor therapy within 30 days prior to death [1]. However, increasing evidence suggests that this treatment approach is neither effective nor useful in most cases [2, 3].

The American Society of Clinical Oncology (ASCO) recommends that end-of-life (EOL) anti-cancer therapy should only be given according to recommended guidelines [4••]. These guidelines recommend stopping anticancer therapy in patients with advanced solid tumors if the performance status (PS) is ECOG (Eastern Cooperative Oncology Group) 3–4, no benefit from prior evidence-based therapies has been observed, the patients are ineligible for clinical trials, or the chance of success is expected to be very low [4••]. Many studies have shown that patients who meet the above criteria do not benefit from antitumor therapy [4••]. Exceptions are patients who, for example, have special disease characteristics (e.g., certain mutations) that predict response to specific therapeutic measures. In any case, supportive and palliative measures should be implemented in patients with advanced metastatic cancer [5]. From the early 1980s, there have been recommendations not to give chemotherapy to patients with a poor ECOG PS (from ECOG 3). In most cases, a poor PS indicates poorer response, shorter survival, and increased toxicity due to chemotherapy [6, 7]. Moreover, there is little evidence of treatment success in patients with poor PS, since many studies reviewing new drugs usually only test patients with a good PS [4••]. 

In most patients with solid metastatic tumors, the chance of treatment success after unsuccessful third-line therapy is very low, but the probability of toxicity is high. Therefore, ASCO guidelines do not recommend further antitumor therapy after an unsuccessful third line of treatment [4••].

The first major report on mortality within 30 days of chemotherapy was conducted by the National Confidential Enquiry into Patient Outcome and Death (NCEPOD) in the UK [8••]. The reported mortality rate within 30 days of SACT was 2% and has been used as the historical benchmark [8••]. Following this report, numerous centers have published data on SACT given within 30 days of death [9–27]. Comparisons between studies are difficult due to differences in the types of tumors included/excluded, treatment with curative/palliative intent, and treatment modality. Furthermore, the studies reported different outcome measures, with most reporting the number of deaths within 30 days of treatment as a proportion of all patients who received treatment and, less frequently, as a proportion of all deaths. After the NCEPOD report, the Christie Cancer Centre in the UK implemented the recommendation to review all deaths within 30 days of SACT at morbidity and mortality meetings and reassess progress through an audit process [8••]. Over a 4-year period, this practice did not reduce the rate of deaths within 30 days of SACT and had a minor, statistically insignificant reduction in the rate of treatment-related deaths [21]. In contrast, Wilson et al. reported two audits conducted at Auckland Hospital 6 years apart [18]. Mortality within 30 days of chemotherapy treatment decreased slightly, with rates of 2.8% in 2009 and 2.2% in 2015. They proposed a series of clinical interventions to inform this improvement implementation plan [18]. An Australian study examined the use of aggressive treatments, including SACT, in the last 30 days of life while considering new treatments like immune checkpoint inhibitors. The authors proposed a quality improvement plan that has been shown to increase the use of palliative care referrals and reduce the use of SACT [28].

Despite the limited evidence for the efficacy of therapy beyond third line, this is not infrequently administered. At a large university facility in Michigan, as many as 50% of all patients with solid tumors received chemotherapy in the last 2 weeks of life [29].

A study published in 2014 was able to show that patients with metastatic cancer and poor general condition who were still receiving chemotherapy in the last months of life were much more likely to undergo intensive care measures (ventilation, resuscitation, or both) than EOL patients no longer receiving chemotherapy [30]. In addition, chemotherapy patients were transferred to a hospice very late or died less frequently at home [30]. Both the intensive medical measures and late transfer to hospice are associated with a poorer QOL at EOL [31]. It is not appropriate to draw a general conclusion that chemotherapy is futile for seriously ill patients in advanced stages. Nevertheless, it is crucial to consider that purely palliative symptom control in the sense of “best supportive care” can be a favorable alternative for numerous patients.

There have been significant recent developments in the treatment of cancer with many new therapy options demonstrating clinical evidence for improved survival and QOL. SACT includes cytotoxic chemotherapy, endocrine or hormonal agents, targeted or biologic agents, and immune checkpoint inhibitors. Non-chemotherapy treatments are often associated with simpler routes of administration, less but not negligible adverse effect profiles, and the potential of profound and durable clinical responses. This has made the decision-making process for commencing, continuing, and ceasing SACT more complex and requires a careful consideration of key factors, specifically disease biology, patient and family expectations, and clinician bias.

The guidelines of ASCO explicitly mention “cancer-directed therapies,” which include anti-tumor treatments, and not exclusively chemotherapy [4••]. However, the recommendations are derived almost exclusively from chemotherapy studies. Compared to conventional chemotherapy, fewer studies have investigated the appropriate time to discontinue treatment with new, so-called targeted drugs, such as tyrosine kinase inhibitors or antibodies, based on the above criteria. Furthermore, new oral therapies are initiated more quickly and terminated later.

A systematic review of the literature evaluated a total of 49 studies relating to anticancer therapy at the EOL [32•]. None of the studies provided criteria for stopping oral anticancer therapy. In most cases, therapy was discontinued when intolerable side effects occurred. However, the personal experience of the treating physicians with the respective treatments also influenced the continuation of the therapy. In some studies, the decision to terminate the therapy was made by the patient. In other studies, the decision to continue the therapy was made by the physicians based on the success of the therapy. The literature review demonstrated an imperative necessity for precise guidelines regarding the termination of anti-cancer therapy, and current research is inadequate regarding the new generation of drugs such as tyrosine kinase inhibitors (TKIs) [32•]. Studies to determine criteria for the duration of therapy are necessary for these new therapeutic measures, especially oral antitumor therapies [33].

A recent comprehensive literature search was conducted to find relevant publications on chemotherapy cessation [34•]. The authors retrieved a total of 2700 records, and 141 were identified as eligible for inclusion in their review. Staples et al. found that palliative chemotherapy did not achieve the goal of tumor-related symptom reduction for patients who had experienced progressive disease with more than two prior lines of chemotherapy [34•].

**Fig. 1:**
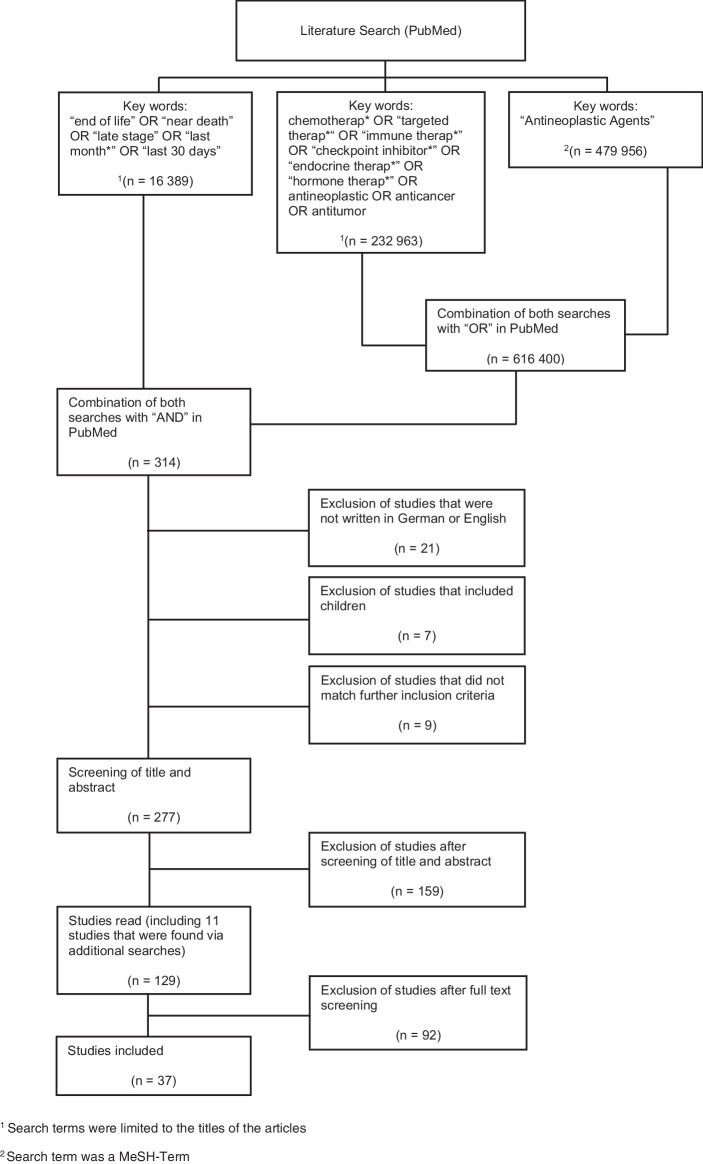
Flow chart of literature research: The data that support the findings of this review article are available upon request. See supplement

In our own review, we identified 37 studies as relevant for a narrative review (Fig. 1; see supplement). Table 1 includes studies which evaluate the impact of SACT on cancer patients receiving therapy in the last weeks of life (Table 1). Table 2 includes studies which report on predictive factors that are associated with the receipt of SACT at the EOL of cancer patients (Table 2).

**Table 1:** Impact of systemic anticancer treatment in selected clinical studies

Reference	Sampling interval	Type of study	Sample characteristics	Results/impacts	Cut-off
Bao et al	2006–2011	Retrospective cohort study	3825 patients that were at least 66 years old when receiving their pancreatic cancer (stage IV) diagnosis and that died within the next 12 months after receiving their diagnosis; of those, 749 received chemotherapy within the last 30 days of life	More often experienced inpatient admissions to hospitals, more often admitted to emergency departments, more often died in hospitals, lower median number of the days of receiving hospice care, increase in out-of-pocket costs for medical care	Last 30 days
Beaudet et al	1 November 2014–31 October 2016	Retrospective analysis of medical records	90 decedents with metastatic NSCLC that received at least one line of palliative SACT; of those, 20 patients received SACT in the last 30 days of life	Less often involvement of palliative care team before death, more often died in a hospital and less often died in hospices or at home, less often received palliative sedation or medical aid in dying, shorter median OS	Last 30 days
Hiramoto et al	August 2011–August 2016	Retrospective analysis of medical records	300 cancer patients of which 50 received chemotherapy within 30 days before death	More often experienced nausea and vomiting, had a higher mean hydration (in L/day)	Last 30 days
Mallet et al	January 2015–July 2017	Retrospective analysis of medical records	582 cancer patients that died within the study period; of those, 338 received SACT in the last 12 weeks of life and 128 received SACT in the last 4 weeks of life	More often admissions to acute hospitals, more often died in hospitals	Last 12 weeks and last 4 weeks
Mathew et al	1 January 2010–30 September 2014	Retrospective cohort study	274 decedents with metastatic breast cancer; of those, 62 patients received chemotherapy in the last 4 weeks of life	Shorter median OS	Last 4 weeks
Mohammed et al	1 January 2010–30 September 2014	Retrospective cohort study	420 decedents with advanced non-hematological cancers; Of those, 124 received chemotherapy in the last month of life	More often died in critical care units	Last 30 days*
Näppä et al	2008	Retrospective analysis of medical records	374 decedents, with epithelial cancer, that were treated with palliative chemotherapy; of those, 87 received chemotherapy in the last month of life	More often admissions to hospitals within 30 days after receiving the last palliative chemotherapy, more admissions to hospitals due to reasons related to chemotherapy within 30 days after receiving the last palliative chemotherapy, less documented decisions about chemotherapy cessation and shorter median times from the decision to death, more deaths in hospitals, less deaths at home or in nursing homes	Last 30 days*
Lapeyre-Prost et al	2014	Retrospective analysis of medical records	437 decedents with metastatic gastrointestinal cancer; of those, 293 patients received chemotherapy in the last 3 months of life and 121 patients received chemotherapy in the last month of life	Impact (last 3 months): Received more often parenteral nutrition within the last 3 months of life Impacts (last month): less often experienced interventions of the palliative care team, shorter hospital stays in the last 3 months of life, more often died at a gastrointestinal oncology hospital unit, less often died at home or in a palliative care unit, more often died because of reasons not related to cancer progression and more often died due to other acute reasons, experienced shorter periods of time between the admission to palliative care units and death, had a shorter median OS	Last 3 months and last 30 days*
Saito et al	1991–1999	Retrospective analysis of medical records	7879 Medicare-enrolled cancer patients that were at least 65 years old and who survived ≥ 3 months after receiving the advanced NSCLC diagnosis; of those, 299 patients still received chemotherapy in the last 14 days of life	More hospital deaths, more ICU admissions within the last month of life, more often > 1 ER visit within the last month of life, more often a missing hospice admission or more often a hospice stay for ≤ 3 days	Last 3 months and last 30 days*
Schulkes et al	February 2011–August 2015	Retrospective analysis of medical records	604 cancer decedents that received palliative chemotherapy and of which 300 received chemotherapy in the last 3 months of life	More unexpected hospitalizations, more ER visits, more often died in hospitals, less often died at home	Last 3 months
Sheng et al	2010–2014	Retrospective analysis of medical records	3350 patients that died of advanced solid tumors; of those, 387 received chemotherapy in the last month of life	Shorter median OS, more often admitted to ICU within the last month of life, more ER visits, received CPR more often, received invasive ventilation support more often, more likely to die in a hospital	Last 3 months
Tsai et al	2005–2012	Retrospective case–control study	42,678 NSCLC decedents of which 3439 received targeted therapy within the last 30 days of life	Patients more often lacked hospice referral and had higher chances for > 1 emergency department visit, hospitalizations for > 14 days, ICU admissions, intubation/mechanical ventilation, receiving CPR	Last 30 days
Wu et al	2009–2011	Retrospective analysis of medical records	49,920 patients that died with metastatic cancer; of those, 8098 received chemotherapy within the last 2–6 months of life	More often > 1 ER visits, more ICU admissions, less often > 1 hospital admission, more often endotracheal intubation, received less often hospice care, survived less often more than 6 months after diagnosis	Last 2–6 months
Zhu et al	2010–2016	Retrospective cohort study	143 SCLC decedents, of which 44 patients received chemotherapy in the last two month of life and 23 received chemotherapy in the last month of life	Impacts (last 2 months): shorter median OS Impacts (last month): more often increases in the ECOG PS score, more ICU admissions and ER visits in the last month of life, more often experienced deaths in hospitals, more often received invasive ventilation support, more often experienced CPR, shorter median OS	Last 2 months and last 30 days*

*CPR* cardiopulmonary resuscitation, *ECOG PS* Eastern Cooperative Oncology Group Performance Status, *ER* emergency room, *ICU* intensive care unit, *NSCLC* non-small cell lung cancer, *OS* overall survival, *SACT* systemic anticancer therapy, *SCLC* small cell lung cancer

^*^Referred by the authors as “Last month of life”

**Table 2:** Predictive factors for receiving systemic anticancer treatments in selected clinical studies

Reference	Sampling interval	Type of study	Sample characteristics	Predictive factors for SACT in the last days of life	Cut-off
Allen et al	2005–2014	Retrospective analysis of medical records	16,501 lung cancer decedents of which 1474 received chemotherapy in the last month of life 4144 pancreatic cancer decedents of which 477 received chemotherapy in the last month of life	Lung cancer: male gender, younger age, regional or distant metastases, high socioeconomic status Pancreatic cancer: male gender, younger age (when comparing < 50 and > 70 aged patients), distant metastases, high socioeconomic status	Last 30 days
Batra et al	2011–2016	Retrospective population-based study	511 patients with metastatic colorectal cancer that received chemotherapy and of which 132 initiated chemotherapy within the last 90 days of life	ECOG PS > 1, lower CCI score	Last 90 days
Bloom et al	1 April 2015–1 April 2019	Retrospective review of medical records	97 patients had ICI treatment in their end-of-life stage over 4 years. 40% received 1 dose within the last 30 days before death	50% + had ECOG PS of 2 or higher, 17% had PS of 3. Over 60% were hospitalized, 25% passed away in hospital	Last 30 days
Fang et al	2007–2013	Retrospective review of medical records	147,254 breast, lung, colorectal and prostate cancer decedents that were at least 65 years old; of those, 5.8% received chemotherapy within the last 14 days of life	Lower age at death, married patients, white non-Hispanic patients, lower CCI, more advanced cancer stages, higher performance status score, patients from urban counties, patients with a higher median income, higher education, earlier year of death, breast cancer, lung cancer, tumor grade 4, AJCC stage of 3–4	Last 14 days
Formoso et al	2017–2020	Retrospective population-based study	55,625 cancer patients that died in the time interval, 15.3% received anticancer drugs in the last month of life	Hematologic cancers, younger age, hospital admission in the last 6 months of life, lack of palliative care in the last 30 days of life, no surgery in the last 6 months of life, non-aggressive tumors	Last 30 days
Goksu et al	2010–2011	Retrospective analysis of medical records	373 patients that died from stage IV solid tumors and of which 89 patients received chemotherapy in the last month of life	Age < 65 years, ECOG PS score of 2 or 3, newly diagnosed patients	Last 30 days*
Hashimoto et al	2002–2006	Retrospective analysis of medical records	255 cancer decedents that received palliative chemotherapy; Of those, 120 received chemotherapy within 90 days before death	Lack of information about palliative care units, presence of symptoms, age ≤ 45 years	Last 90 days
Hiramoto et al	August 2011–August 2016	Retrospective analysis of medical records	300 advanced cancer patients of which 16 received chemotherapy within 14 days and 50 received chemotherapy within 30 days before death	ECOG PS score ≥ 2, GPS of 2	Last 14 days and 30 days
Hui et al	1 September 2009–28 February 2010	Retrospective analysis of medical records	816 advanced cancer decedents of which 116 received targeted therapy and 147 received chemotherapy within the last 30 days of life	Predictive factors (targeted agents): younger age, hematologic cancers Predictive factors (chemotherapy): hematologic cancers, survival < 6 months between diagnosis and death	Last 30 days
Kajimoto et al	December 2013–August 2020	Retrospective analysis of health insurance claims	5759 cancer patients that died in the study interval; of those, 278 received anticancer treatment in the last 14 days of life	< 60 years of age	Last 14 days
Karim et al	2010–2012	Retrospective review of medical records	115 cancer decedents of which 41 received chemotherapy within the last 60 days of life	ECOG ≤ 3, lack of palliative care	Last 60 days
Edman Kessler et al	2010–2015	Retrospective cohort study	Greek cohort: 477 metastatic breast cancer decedents of which 222 patients received chemotherapy within the last month of life Swedish cohort: 1358 metastatic breast cancer decedents of which 315 patients received chemotherapy within the last month of life	Predictive factors (Greek cohort): no significant predictors mentioned Predictive factors (Swedish cohort): younger age, higher albumin levels	Last 30 days*
Liu et al	2001–2006	Retrospective population-based study	204,850 cancer decedents of which 17.5%, 17.4%, 17.3%, 19.0%, 20.0%, and 21.0%, for each study year respectively, received chemotherapy in the last month of life	Male gender, younger age, being single, lower CCI, breast cancer, hematological cancers, presence of metastases, survival 3–12 months after diagnosis, having an oncologist as a primary physician, being patient in a teaching hospital	Last 30 days*
Massa et al	1 January 2007–30 June 2014	Retrospective analysis of medical data	365 decedents with metastatic colorectal cancer of which 26 received chemotherapy within the last 14 days of life	Age ≤ 70 years, patients that did not experience any previous line of advanced treatments	Last 14 days
Mathew et al	1 January 2010–30 September 2014	Retrospective cohort study	274 metastatic breast cancer decedents of which 62 received chemotherapy within 4 weeks before death	Younger age at diagnosis of metastatic breast cancer, number of metastatic organ systems involved	Last 4 weeks
Mattsson et al	2010–2015	Retrospective cohort study	42,377 patients that died from cancer and of which 6748 patients received one or more anticancer treatments in the last 30 days of life	Male gender, younger age, breast cancer, melanoma, prostate cancer, CCI of 0, cancer diagnosis within last 6 months of life	Last 30 days
Mieras et al	1 June 2013–31 July 2015	Retrospective patient file study	1322 metastatic lung cancer decedents of which 232 received anticancer treatment in the last month of life	Predictive factors (chemotherapy): SCLC, ≥ 3rd-line treatment Predictive factors (TKIs): NSCLC, 2nd-line treatment, prescribing oncologist younger than 51 years	Last 30 days*
Randén et al	2009	Retrospective analysis of medical records	346 decedents with disseminated cancer of which 32% received anticancer treatments within the last month of life	Younger age, high level of education	Last 30 days*
Robausch et al	2012–2016	Retrospective analysis of medical records	80,818 decedents with cancer. Regarding cancer treatment during the last 30 days of life, 6.9% of patients received chemotherapy, 1.7% received radiation therapy, and 0.75% received monoclonal antibody treatment	Slight variations could be observed comparing the Austrian federal states Salzburg had the highest proportion of cancer patients who received chemotherapy within a month before death (10.4%), while Vorarlberg (5.3%) and Burgenland (5.4%) had the lowest	Last 30 days
Rochigneux et al	2010–2013	Retrospective register-based study	279,846 decedents with solid metastatic cancers of which 19.5% received chemotherapy in the last month of life	Male gender, lower age, lower amounts of Charlson comorbidities, chemosensitive tumors, skin cancer, head and neck cancer, tumors of the female genital organs, breast cancer, tumors of the respiratory system, patients of comprehensive cancer centers, patients of private hospitals, patients in rehabilitation care facilities, higher annual volume of chemotherapy, lack of a palliative care unit	Last 30 days*
Sheng et al	2010–2014	Retrospective analysis of medical records	3350 patients that died of advanced solid tumors; Of those, 387 received chemotherapy in the last month of life	≥ 56 years of age, cancer type, metastatic disease, high education level, two or more lines of treatment, patients of general hospitals	Last 30 days*
Tsai et al	2005–2012	Retrospective case–control study	42,678 NSCLC decedents of which 3439 received targeted therapy within the last 30 days of life	Younger age, late disease stage, adenocarcinomas, survival for > 6 months after diagnosis, later year of death, having a treating pulmonologist, having a treating oncologist, having a younger treating physician, having a treating physician with higher lung cancer case volumes, being treated in district or non-public hospitals, being treated in teaching hospitals, being treated in hospitals with higher lung cancer case volumes	Last 30 days
Yun et al	1 January 2004–31 December 2004	Retrospective analysis of medical records	3750 cancer decedents of which 43.9% received chemotherapy in the last 3 months and 30.9% received chemotherapy in the last month of life	Predictive factors (Last 3 months): younger age, female gender, high income level, small hospital size, part of Medicaid program, lack of hospice facility, responsive cancer types Predictive factors (last month): younger age, female gender, marriage, high income level, small hospital size, lack of hospice facility, responsive cancer types	Last 3 months and last 30 days*
Zdenkowski et al	2009–2011	Retrospective chart review	1131 patients that received palliative chemotherapy; Of those, 138 patients died within 30 days of receiving palliative chemotherapy	Male gender, receiving palliative care less than 30 days	Last 30 days
Zhang et al	April 2007–June 2019	Retrospective analysis of medical records	605 metastatic cancer decedents of which 98 patients were treated with palliative chemotherapy in the last month of life	Age ≤ 50 years	Last 30 days*

*AJCC* American Joint Committee on Cancer, *CCI* Charlson Comorbidity Index, *ECOG PS* Eastern Cooperative Oncology Group Performance Status, *GPS* Glasgow Prognostic Score, *NSCLC* Non-small cell lung cancer, *SCLC* small cell lung cancer, *TKI* tyrosine kinase inhibitor

^*^Referred by the authors as “Last month of life”

### The impact of SACT at the EOL (Table 1)

Bao et al. investigated the association between chemotherapy use in patients with stage IV pancreatic cancer and healthcare use, as well as Medicare and out-of-pocket costs in the last 30 days of life [35]. The study concluded that chemotherapy use among older patients diagnosed with metastatic pancreatic cancer leads to increased healthcare utilization and higher patient out-of-pocket costs near death [35]. According to a retrospective study that analyzed the use of palliative SACT for patients with advanced non-small cell lung cancer, 22% of patients received this therapy within 30 days of death. This was associated with reduced access to palliative care, higher rates of in-hospital death, a decreased use of voluntary assisted dying and palliative sedation, and a shorter median overall survival of 4.0 months compared to 9.0 months for patients who did not receive the therapy close to death [36].

Eastern Cooperative Oncology Group Performance Status (ECOG-PS) and Glasgow Prognostic Score were significant prognostic factors regarding the effectiveness of aggressive EOL chemotherapy in a study performed by Hiramoto et al. [13]. Furthermore, patients who died within 30 days after chemotherapy had a higher rate of nausea, vomiting, and hydration [13].

An Irish study found that a significant percentage of patients received treatment in the last 12, 4, and 2 weeks of life, which was associated with more hospital admissions, procedures, and in-hospital deaths [37]. Patients who received chemotherapy were also referred to specialist palliative care services at a later stage than those who did not [37].

A study by Mathew et al. reviewed medical charts of metastatic breast cancer patients who received immune checkpoint inhibitor (ICI) treatment within 30 days of death [38]. Out of 97 patients evaluated over a 4-year period, 40% received only one dose of ICI during that period. Over 50% had a poor ECOG-PS, and more than 60% were hospitalized. Additionally, 65% visited the emergency department, 20% required intensive care unit admission, and 25% died in the hospital [38]. The study’s findings add to the current knowledge of using ICI treatment for advanced cancer patients in their EOL stage [38].

A study analyzing the use of palliative chemotherapy in advanced non-hematological cancer patients revealed that 29.5% of all patients received chemotherapy in the last month before death. There was a strong association between last month before death palliative chemotherapy and place of death (critical care vs. regular ward) as well as mode of admission (emergency room vs. outpatient department vs. direct transferred from other health care facilities) [39].

In a Swedish study, 23% of patients received chemotherapy during their last month of life, leading to shorter survival time, frequent hospital admissions, less documented decisions to cease treatment, and less deaths at home [29].

Among 437 patients with gastrointestinal cancer, 67% received CT within 3 months of death and 28% within 1 month. Patients receiving CT within 1 month had poorer OS and received less palliative care [40].

A study by Saito et al. examined the impact of continuing chemotherapy on survival and EOL care for 7879 Medicare-enrolled patients aged 65 or older who survived at least 3 months after diagnosis of advanced non-small cell lung cancer [41]. Three different statistical approaches showed no additional survival benefit from continuing chemotherapy within 14 days of death but was associated with a decreased likelihood of receiving hospice care [41].

An investigation of 604 patients of whom 50% received chemotherapy in the last 3 months revealed that these patients had a higher rate of unplanned hospital admissions and emergency room visits [42].

A retrospective study investigating the prevalence and outcomes of EOL chemotherapy in advanced solid cancer patients revealed that of the 3350 decedents included, 5.3%, 11.6%, and 25.0% received EOL chemotherapy within the last 2 weeks, 1 month, and 2 months of life respectively [43]. Receiving EOL chemotherapy was associated with inferior OS, more intensive treatments, and hospital death. However, subgroup analysis suggested that receiving oral agents was associated with better outcomes [43].

A study analyzing the use of targeted therapies in the last month of life for non-small-cell lung cancer patients found that 21.3% of patients received targeted therapy within 30 days of death [44]. Younger patients, patients with adenocarcinoma, longer survival post-diagnosis, and patients treated by respiratory physicians or oncologists, were more likely to receive targeted therapy [44]. According to the results of this study, targeted therapy at EOL should be a quality-of-care indicator [44].

Data of a large Taiwanese trial including 49,920 patients who underwent palliative chemotherapy were analyzed. Palliative chemotherapy was shown to be associated with more aggressive EOL care, including emergency room visits, intensive care unit admissions, endotracheal intubation, and less hospice service at the EOL [45].

Another study analyzed EOL chemotherapy in small cell lung cancer patients and documented an association with shorter survival and more aggressive care [46]. Younger patients were more likely to receive EOL chemotherapy [46].

Altogether, these studies identified several different factors that were related to the impact of SACT on health and healthcare-related outcomes, including an increase in hospital admissions, emergency department visits, in-hospital deaths, and fewer days in hospice care. Some studies demonstrated a decreased use of medical aid in dying and palliative sedation, and a shorter median OS. Patients were referred later to specialist palliative care services. Overall, palliative chemotherapy was associated with more aggressive EOL care, including emergency room visits, intensive care unit admissions, and endotracheal intubation. Some of the studies found that younger patients were more likely to receive EOL chemotherapy.

### Predictive factors associated with SACT at the EOL (Table 2)

According to Allen et al., 9–12% of lung and pancreatic cancer patients received EOL chemotherapy, with a higher prevalence in males with advanced metastatic cancer [47]. This was associated with higher rates of deaths within an acute care facility [47].

In analyzing how often patients with metastatic colorectal cancer started a new chemotherapy regimen within 90 days of death, a Canadian study showed that of the 511 patients who received chemotherapy, 25.8% started chemotherapy near EOL [48]. Factors such as comorbidity index score and oncology PS predicted for initiation of chemotherapy near EOL [48].

A recent study showed that 97 patients had ICI treatment in their EOL stage over 4 years. Forty percent received a single dose within the last 30 days before death [49]. Over 50% had an ECOG-PS of 2 or higher, and 17% had a PS of 3. Hospitalization rate was over 60%, and 25% died in hospital [49].

A large trial analyzed the administration of EOL chemotherapy and targeted therapy in patients with breast, lung, colorectal, or prostate cancer and time of death between 2007 and 2013 [50]. The administration of chemotherapy within 14 days of EOL declined, while it increased within 4 to 6 months of EOL. Targeted therapy use remained stable. Physician-level variation accounted for 5.19% of the variation in 14-day EOL chemotherapy [50]. This study suggests that national benchmarking can effectively reduce EOL chemotherapy use [50].

An Italian study investigated the use of anticancer drugs and palliative care services in EOL care for 55,625 cancer patients who died between 2017 and 2020 [51•]. The study found that the use of anticancer drugs was inversely associated with receiving palliative care services in the last month of life [51•]. The authors suggest that the implementation of adequate prognostic tools is key to improve the appropriateness of EOL care [51•].

A Turkish study examined the use of palliative chemotherapy near the EOL [52]. Of the 373 patients who died from stage IV solid tumors between 2010 and 2011, 23.9% received chemotherapy in the last month of life, and 10.5% received chemotherapy in the last 14 days [52]. Age, recent diagnosis, and PS influenced the likelihood of receiving chemotherapy in the last month of life [52].

An earlier study analyzed 255 patients who received palliative chemotherapy and died between 2002 and 2006 [53]. Lack of information about palliative care units, presence of symptoms, and age ≤ 45 years were predictive factors for receiving palliative chemotherapy in this study [53].

A retrospective analysis of 300 patients from 2011 to 2016 identified ECOG-PS and GPS as significant prognostic factors at the last administration of chemotherapy [13]. Within 14 and 30 days, 5.3% and 16.7%, respectively, died. Those with PS 2–4 and GPS 2 had median survival of 38 days. A higher prevalence of nausea, vomiting, and hydration was found in those who died within 30 days [13].

In a study by Hui et al., predictive factors for targeted agents were younger age and hematologic cancers, whereas predictive factors for chemotherapy were hematologic cancers and survival < 6 months between diagnosis and death [54].

Out of 5759 patients who died of cancer between 2013 and 2020 in Japan, 4.8% received anticancer therapy within 14 days of death. Patients aged 60 and above had a higher probability of receiving anticancer therapy near EOL [55].

In a retrospective study of adult cancer patients who died in a hospital, the authors investigated factors associated with a shorter time from last chemotherapy to death (TLCD) [56]. Patients with better PS and under a medical oncology service had a shorter TLCD, while those with no palliative care involvement had a higher risk of dying from treatment-related complications and receiving more aggressive EOL care [56]. Palliative care involvement was associated with longer TLCD [56].

In a Swedish study, age and albumin levels were associated with chemotherapy use. This study concluded that EOL chemotherapy may harm patient QOL [57].

A Taiwanese study revealed that factors such as male gender, younger age, lower comorbidity levels, certain cancer types, and being cared for by a medical oncologist or in a teaching hospital were associated with higher rates of continued chemotherapy. Regional healthcare resources did not have an impact on chemotherapy continuation [58].

A study assessing care quality for metastatic colorectal cancer patients revealed that 9.8% started new treatment in the last 30 days of life. Age and no advanced treatment were linked to overuse of SACT [14]. Mathew et al. found that younger age at diagnosis of metastatic breast cancer and the number of metastatic organ systems involved were related to application of SACT at the EOL [38].

A retrospective cohort study found that male gender, younger age, breast cancer, melanoma, prostate cancer, and cancer diagnosis within the last 6 months of life were associated with SACT in the last 30 days of life [59].

An interesting finding was described by Mieras et al. who found that a predictive factor for tyrosine kinase inhibitors (TKIs) for NSCLC was the age of the prescribing oncologist (< 51 years) [60].

Younger age and high level of education was found to be a predictive factor for the prescription of SACT in the last 30 days of life [61].

In Austria, there were slight variations in cancer treatments during hospital stays within 30 days before death, with the highest proportion of patients receiving chemotherapy in the state of Salzburg (10.4%) and the lowest in Vorarlberg (5.3%) and Burgenland (5.4%) [62••].

A very large retrospective register study analyzed data from 279,846 patients with solid metastatic cancers and found that 19.5% received chemotherapy in the last month of life [63]. Factors associated with this included male gender, younger age, lower comorbidities, and having chemosensitive tumors or cancers of the skin, head and neck, female genital organs, or respiratory system. Patients treated at comprehensive cancer centers, private hospitals, or rehabilitation care facilities, and those receiving a higher annual volume of chemotherapy, were also more likely to receive chemotherapy [63]. The lack of a palliative care unit was also associated with receiving chemotherapy in the last 30 days of life [63].

Sheng et al. found that ≥ 56 years of age, cancer type, metastatic disease, high education level, two or more lines of treatment, and patients of general hospitals were associated with SACT at the EOL [43].

Several factors were found to be predictive of receiving chemotherapy in the last 30 days of life in a large retrospective analysis, including younger age, late disease stage, adenocarcinomas, longer survival after diagnosis, later year of death, having a treating pulmonologist or oncologist, younger treating physician, higher lung cancer case volumes of treating physicians, and being treated in district or non-public hospitals, teaching hospitals, or hospitals with higher lung cancer case volumes [44].

A retrospective analysis identified younger age, female gender, marriage, high income level, small hospital size, lack of hospice facility, and responsive cancer types as predictive factors to receive SACT in the last month of life [64]. Male gender was a predictive factor for receiving SACT in a study by Zdenkowski et al. [24].

A retrospective analysis of medical records of 605 metastatic cancer decedents of which 98 patients were treated with palliative chemotherapy in the last month of life found an association of age > 50 years with application of SACT in the last 30 days of life [65].

Altogether, the selected studies found various predictive factors that were linked to enhanced use of SACT at the EOL including sex, younger age, lower comorbidity levels, higher level of education of patients, and even age of the prescribing oncologist (< 51 years). Other factors such as poor PS, high ECOG, or claim of palliative care services were correlated to reduced administration of SACT at the EOL.

## Conclusion

Many studies recommend physicians to prioritize improving QOL and consider discontinuing chemotherapy to direct patients towards early palliative care [66•, 67, 68, 69••]. In the present narrative review, we found a great number of studies supporting the recommendations of the current guidelines by ASCO and ESMO not to use SACT at the EOL and describing reasons and factors hindering good care at the EOL by administering SACT despite current guidelines [4••, 69••].

Patients should be encouraged to enroll in hospice for better EOL palliative care, and oncologists should discontinue chemotherapy as death approaches. This indicates a higher consumption of healthcare services, raising concerns about the benefits of palliative chemotherapy for terminally ill cancer patients.

Palliative SACT is a common treatment approach for patients with advanced cancer. It aims to alleviate symptoms, improve QOL, and extend survival. However, it is important to balance the potential benefits of treatment with its side effects and impact on patients’ EOL experiences. Physicians and patients should discuss the goals of treatment and consider stopping or modifying SACT as appropriate [70]. Timely integration of palliative care services can help patients and families manage symptoms, make informed decisions, and plan for EOL care and should be routinely implemented in the oncologist’s care plan [71, 72]. Reducing futile treatment at the EOL and implementing timely palliative care can improve patients and their caregivers’ QOL substantially.

## Abbreviations

ASCO: American Society of Clinical Oncology
ECOG: Eastern Cooperative Oncology Group
ECOG-PS: Eastern Cooperative Oncology Group Performance Status
EOL: End of life
ESMO: European Society of Medical Oncologists
ICI: Immune checkpoint inhibitor
NCEPOD: National Confidential Enquiry into Patient Outcome and Death
OS: Overall survival
PS: Performance status
QOL: Quality of life
SACT: Systemic anticancer therapy
TLCD: Time from last chemotherapy to death
